# Recombinant Human Growth Hormone Inhibits Lipotoxicity, Oxidative Stress, and Apoptosis in a Mouse Model of Diabetic Cardiomyopathy

**DOI:** 10.1155/2021/3899356

**Published:** 2021-12-09

**Authors:** Zuowei Pei, Xiang Wang, Chenguang Yang, Min Dong, Fang Wang

**Affiliations:** ^1^Department of Cardiology, Beijing Hospital, National Center of Gerontology, Institute of Geriatric Medicine, Chinese Academy of Medical Sciences, Beijing 100730, China; ^2^School of Life Science, University of the Chinese Academy of Sciences, Beijing 100049, China; ^3^Graduate School of Peking Union Medical College, Chinese Academy of Medical Science, Beijing 100730, China

## Abstract

Recombinant human growth hormone (rhGH), widely used in clinical studies, exerts protective effects against cardiac damage. Here, we investigated the effects and mechanisms underlying the effects of rhGH on cardiac functions in db/db mice. C57BL/6J and db/db mice were subjected to rhGH treatment. Metabolic parameters, cardiac function and morphology, oxidative stress, lipid metabolism, and apoptosis were evaluated 16 weeks after rhGH treatment. Although rhGH did not significantly affect fasting blood glucose levels in db/db mice, it protected against diabetic cardiomyopathy, by improving cardiac function and reducing oxidative stress in the heart. In addition, rhGH treatment exhibited anti-apoptotic effects in the heart of db/db mice. The rhGH treatment, besides inhibiting oxidative stress and apoptosis, ameliorated cardiac dysfunction by inhibiting lipotoxicity in mice with type 2 diabetes. These findings suggest that rhGH is a promising therapeutic agent for diabetic cardiomyopathy.

## 1. Introduction

Unlike healthy individuals, patients with diabetes are 2-3-fold more likely to develop cardiovascular disease and have a higher risk of developing myocardial infarction, heart failure, or stroke [1, 2]. Once the cardiovascular disease develops, patients with diabetes have a significantly worse prognosis than those without diabetes. Diabetic cardiomyopathy (DCM) is a serious diabetic cardiovascular complication, characterized by cardiac structural remodeling and dysfunction, and is related to various pathological conditions, including myocardial lipotoxicity, oxidative stress, glucose (Glu) toxicity, cellular apoptosis, autophagy, and Fundc1-dependent mitophagy [3–8]. Mitochondrial fission and mitophagy are involved in organ damage observed under diabetic condition [9]. A metabolic switch, characterized by a change from fatty acids to glucose as the preferred substrate, is a characteristic feature of cardiac dysfunction [10]. Currently, lipotoxicity and oxidative stress are believed to be the two main factors contributing to the pathogenesis of DCM induced by type 2 diabetes mellitus (T2DM) [11]. However, effective strategies to prevent or improve cardiac damage in patients with diabetes are yet to be devised.

Growth hormone (GH) is a 191-amino acid peptide naturally released from the anterior pituitary gland; in humans, it plays an important role in regulating metabolism and body composition [12]. Growth hormone is an effective anabolic agent that can reverse nutritional and metabolic abnormalities associated with severe catabolic states [13, 14]. Previous studies have shown that GH can improve metabolism and exert various effects, including antiapoptotic and anti-inflammatory effects [15, 16]. Growth hormone has been shown to alleviate myocardial fibrosis [17, 18], reduce the inflammatory response in the peri-infarct region after myocardial infarction [19, 20], and improve cardiac function in animal models [21, 22]. It has also been examined as a potential adjunctive therapeutic agent in patients with remote myocardial infarction and heart failure [23, 24]. Recombinant human GH (rhGH) can be synthesized *in vitro* using gene recombination technology and exhibits the same characteristics and physiological effects as GH secreted by the human body. It also enhances resistance, regulates metabolic substances, and reduces inflammation [25, 26]. Some reports have indicated that rhGH does not promote the proliferation of cancer cells. European Medical Agency (EMA) presented safety and effectiveness data for treatment of adults and children up to 10 years of age with rhGH and reported no severe adverse events (AEs) of diabetes, impaired glucose tolerance, or malignancy [27, 28]. Although several beneficial effects of rhGH have been reported, its action mechanisms remain poorly understood. Here, we investigated the effects of GH treatment on DCM in db/db mice.

## 2. Materials and Methods

### 2.1. Animal Model and Treatment

Twelve-week-old C57BL/6J and db/db male mice were purchased from Shanghai Model Organisms Center (Shanghai, China). C57BL/6J mice were used as controls and db/db mice were used to construct a model of T2DM with obesity. We divided the mice into four groups, with eight animals per group: C57BL/6J, control group; C57BL/6J+rhGH (3 IU/kg/day), the rhGH group; db/db group; and db/db+rhGH (3 IU/kg/day) group. We used the dose of rhGH as previously described [27, 29, 30]. The mice were injected with recombinant human growth hormone (rhGH; GenSci.LT, Changchun, China) once daily. All mice were provided water and chow diet *ad libitum* throughout the experimental period, and the body weight was measured every week. All mice were maintained under a 12 h light/dark cycle at 20-22°C. All mice were fasted for 12-16 h before sampling and testing. Our study lasted 16 weeks, and at the end of the study, the mice were anesthetized (pentobarbital 40 mg/kg, intraperitoneally) and blood samples were collected from the eye sockets; then, the mice were euthanized with a high dose of pentobarbital (100 mg/kg, intraperitoneally); lack of breathing and heartbeat was used as an indicator of death. Serum was obtained by centrifugation from blood samples and stored at -80°C until further analysis; heart tissues were collected from the mice, and heart weight was determined. Some part of the heart tissues was fixed and embedded in paraffin for the subsequent analyses, while the remaining tissue was snap-frozen in liquid nitrogen for immunoblotting analyses. All studies involving animal experimentation followed the National Institutes of Health Guidelines on the Care and Use of Animals.

### 2.2. Echocardiography

After 16 weeks of treatment, echocardiography was performed using the Vevo 2100LT microultrasound system (FUJIFILM VisualSonics, Inc., Ontario, Canada). The mice were anesthetized with 1.5% isoflurane and immediately placed on a 37°C thermostat to maintain normal body temperature; the position and direction of the ultrasound beam were slowly adjusted to obtain echocardiography images of the left ventricle. M-mode images were acquired to evaluate the left ventricular function parameters.

### 2.3. Serological Tests

Sera were obtained by centrifuging the clotted blood collected from the eye sockets of the mice and stored at -80°C. Serum triglyceride (TG), total cholesterol (TC), lactate dehydrogenase (LDH), creatine kinase MB (CK-MB), and blood Glu levels were examined using commercial reagent kits (Nanjing Jiancheng Bioengineering Institute, Nanjing, China).

### 2.4. Cardiac Oxidative Stress Analysis

The heart tissue was macerated with saline solution at a ratio of 1 : 9 mg/*μ*L to yield a homogenate, which was centrifuged for 5 min at 7,000 rpm. The supernatants were used for measuring GSH-Px and MDA using the respective kits (Nanjing Jiancheng Bioengineering Institute), following the manufacturer's instructions.

### 2.5. Histological Staining

The hearts were fixed by perfusion with 10% buffered formalin. Next, the hearts were fixed overnight at room temperature (24°C-26°C), transferred to 70% ethanol, and then embedded in paraffin. Paraffin-embedded tissue slices were deparaffinized via immersion in xylene (three times, 5 min each) and rehydrated in a series of graded alcohol solutions (100%, 90%, 80%, and 70% alcohol, 5 min each). Histological changes were detected by staining the sections with hematoxylin and eosin (HE), FITC-conjugated wheat-germ agglutinin (WGA), Masson's trichrome, and Periodic Acid-Schiff (PAS) stain. Images were acquired using an upright light microscope (Olympus, Tokyo, Japan).

### 2.6. Immunohistochemistry

For immunohistochemical staining, the heart sections were deparaffinized and rehydrated. Next, the sections were blocked with 3% H_2_O_2_ in methanol for 15 min to inactivate the endogenous peroxidases and incubated overnight at 4°C with the primary antibodies: CD36 (rabbit anti-CD36 antibody, 1 : 200; Proteintech, Wuhan, China), SCD-1 (rabbit anti-SCD-1 antibody, 1 : 200; Abcam, England), PGC1-ɑ (rabbit anti-PGC1-ɑ antibody, 1 : 200; Proteintech), CPT-1 (rabbit anti-CPT-1 antibody, 1 : 200; Proteintech), p-AMPK (rabbit anti-p-AMPK antibody, 1 : 100; Proteintech), PPAR-*α* (rabbit anti-PPAR-*α* antibody, 1 : 50; Proteintech), BAX (rabbit anti-BAX antibody, 1 : 200; Proteintech), Bcl-2 (rabbit anti-Bcl-2 antibody, 1 : 200; Proteintech), and cleaved caspase-3 (rabbit anti-cleaved caspase-3 antibody, 1 : 400; Cell Signaling Technology, USA). The sections were incubated with goat anti-rabbit HRP secondary antibody (Histofine Simple Stain Kit; Nichirei, Tokyo, Japan) for 30 min at room temperature. All sections were examined using an Olympus light microscope (Olympus, Tokyo, Japan).

### 2.7. BODIPY Staining

Frozen cardiac tissues were embedded using an optimal cutting temperature (OCT) embedding agent. Next, 6 *μ*m thick sections were cut using a freezing microtome for BODIPY staining. Briefly, sections were immersed in BODIPY solution for 60 min at 25°C, followed by PBS washes. DAPI staining was carried out for 10 min in the dark at room temperature. Next, the slices were washed with PBS three times for 5 min each and sealed with an antifluorescence quenching sealant. Fluorescence microscopy was performed in a dark room for observation and acquiring images.

### 2.8. Reactive Oxygen Species (ROS) Analysis

The heart tissues were embedded with an OCT embedding agent. Next, 6-*μ*m-thick sections were cut using a freezing microtome for ROS staining. Frozen sections were fixed at room temperature with 4% PFA for 5 minutes and washed three times with PBS for 1 minute each. Then, a tissue pen was used to draw a circle around the sections followed by addition of 5 *μ*M 20,70-dichlorofluorescein diacetate (DCFH-DA) to the circled area. The sections were incubated at 37°C for 20 min in dark, followed by PBS washes. DAPI staining was carried out at room temperature for 10 min in the dark. Slices were then washed with PBS three times for 5 min each. The slices were sealed with an antifluorescence quenching sealant, and fluorescence microscopy was performed in a dark room for observation and acquiring images.

### 2.9. TUNEL Staining

The heart tissues were embedded in paraffin and serially sectioned to 5 *μ*m thickness. The sections were deparaffinized and hydrated in xylene and graded series of ethanol and then incubated with proteinase K (37°C, 22 min). The sections were stained using the Fluorescein TUNEL Cell Apoptosis Detection kit (Servicebio Technology Co., Ltd., Wuhan, China). All images were captured using a fluorescence microscope. The cells positive for TUNEL staining and DAPI staining were considered apoptotic cells and counted.

### 2.10. Western Blotting

Proteins were extracted from the heart samples using radioimmunoprecipitation assay buffer (P0013B; Beyotime, Shanghai, China). First, protein samples were separated by 10% sodium dodecyl sulfate-polyacrylamide gel electrophoresis (SDS-PAGE) and then transferred onto polyvinylidene fluoride (PVDF) membranes (Immobilon, Millipore, Billerica, MA, USA). The membranes were blocked with 5% skim milk in TBST buffer (TBS containing 0.1% Tween-20) at room temperature for 1 h and incubated with primary antibody at 4°C overnight. The primary antibodies were against ANP (rabbit anti-ANP antibody, 1 : 500; Invitrogen, USA), BNP (rabbit anti-BNP antibody, 1 : 500; Invitrogen), CD36 (rabbit anti-CD36 antibody, 1 : 1000; Proteintech), SCD-1 (rabbit anti-SCD-1 antibody, 1 : 1000; Abcam), PGC1-ɑ (rabbit anti-PGC1-ɑ antibody, 1 : 1000; Proteintech), CPT-1 (rabbit anti-CPT-1 antibody, 1 : 1000; Proteintech), p-AMPK (rabbit anti-p-AMPK antibody, 1 : 1000; Proteintech), NRF2 (rabbit anti-NRF2 antibody, 1 : 500; Proteintech), HO-1 (rabbit anti-HO-1 antibody, 1 : 1000; Abcam), PPAR-*α* (rabbit anti-PPAR-*α* antibody, 1 : 500; Proteintech), SOD (rabbit anti-SOD antibody, 1 : 2000; Proteintech), NOX4 (rabbit anti-NOX4 antibody, 1 : 1000; Proteintech), CAT (rabbit anti-CAT antibody, 1 : 1000; Proteintech), BAX (rabbit anti-BAX antibody, 1 : 5000; Proteintech), Bcl-2 (rabbit anti-Bcl-2 antibody, 1 : 2000; Proteintech), cleaved caspase-3 (rabbit anti-cleaved caspase-3 antibody, 1 : 1000; Cell Signaling Technology), t-caspase-3 (rabbit anti-t-caspase-3 antibody, 1 : 1000; Proteintech), and anti-*β*-actin (1 : 1000; Proteintech). After washing, the membranes were incubated with the appropriate secondary antibody (anti-rabbit Ig-G, 1 : 2000; Proteintech) for 1 h. The blotted proteins were quantified using the NIH ImageJ software. *β*-Actin was used as the internal control. The protein levels are expressed as protein/*β*-actin ratios.

### 2.11. Statistical Analysis

Data are expressed as mean ± SEM. The differences among multiple groups were analyzed using the one-way analysis of variance (ANOVA) and a subsequent Tukey's test. For all statistical comparisons, a *P* value of <0.05 was considered statistically significant. All statistical analyses were performed using Statistical Package for Social Sciences version 23.0 (SPSS, Chicago. IL, USA).

## 3. Results

### 3.1. Metabolic Characterization

**Figure 1:**
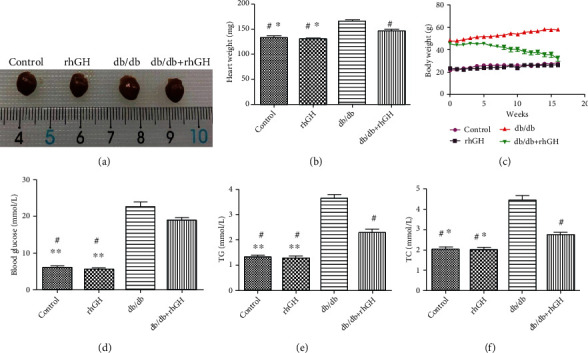
Metabolic data of mice from different groups after rhGH treatment. (a) Representative heart size of mice in different groups. (b) Quantitative analysis of heart weight in different groups. (c) The trend of body weight changes in different groups. (d–f) TC, TG, and Glu levels in different groups. *n* = 8 mice from each group. ^∗^*P* < 0.05 vs. db/db+rhGH group, ^∗∗^*P* < 0.01 vs. db/db+rhGH group, and ^#^*P* < 0.01 vs. db/db group.

The mice in the db/db group showed considerably increased heart weight, body weight, and TC and TG levels, but these values were significantly decreased in the mice in the db/db+rhGH group. However, the rhGH treatment did not significantly decrease the blood Glu levels in db/db mice (Figure 1). Therefore, the protective effects of rhGH in DCM might depend on the regulation of lipid metabolism.

### 3.2. rhGH Improved Cardiac Function and Alleviated Cardiac Tissue Damage in db/db Mice

**Figure 2:**
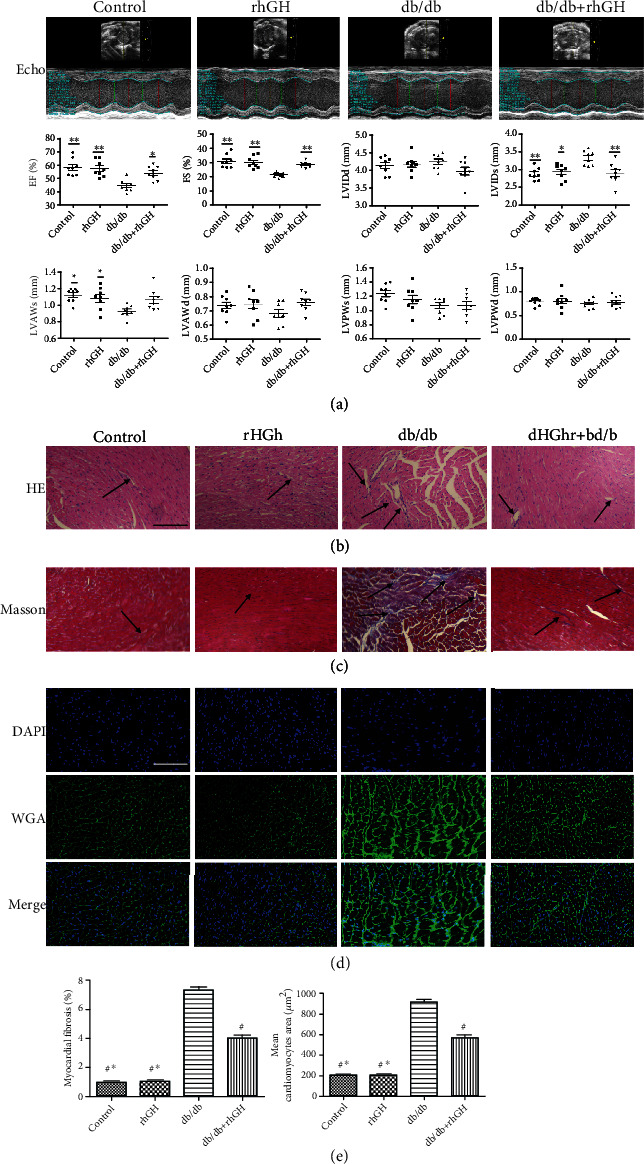
Treatment with rhGH improved cardiac function and alleviated cardiac tissue damage in db/db mice. (a) rhGH improved cardiac function in db/db mice. Typical echocardiogram images are shown in the figure. Quantified of LV ejection fraction (EF), LV fraction shortening (FS), LV internal dimension diastole (LVIDd), LV internal dimension systole (LVIDs), LV anterior wall systole thickness (LVAWs), LV anterior wall diastole thickness (LVAWd), LV posterior wall systole thickness (LVPWs), and LV posterior wall diastole thickness (LVPWd) are shown in the bar graph. *n* = 8 per group. ^∗^*P* < 0.05 vs. db/db group and ^∗∗^*P* < 0.01 vs. db/db group. (b, c) HE staining for tissue structure damage and Masson's trichrome staining for collagen deposition in the cardiac tissue. (d) WGA staining (green fluorescence) and DAPI staining (blue fluorescence) for cardiomyocyte hypertrophy in the cardiac tissue. (e) Quantification of the relative fibrotic area and the cross areas of cardiomyocytes. Scale bar: 100 *μ*m. The arrows indicate positively stained. *n* = 3 per group. ^∗^*P* < 0.05 vs. db/db+rhGH group and ^#^*P* < 0.01 vs. db/db group.

To determine whether rhGH protected the heart against DCM, cardiac function and morphology were assessed. Compared with the control group mice, db/db mice exhibited impaired cardiac function, as evidenced by a decrease in left ventricular ejection fraction and fraction shortening (LVEF and LVFS) and an increase in left ventricular internal dimension (LVID) (Figure 2(a)). With rhGH treatment, cardiac function improved. Hematoxylin and eosin staining revealed inflammatory cell infiltration (Figure 2(b)). Masson's trichrome staining was performed to assess cardiac tissue collagen deposition (Figure 2(c)); WGA staining revealed cardiomyocyte hypertrophy (Figure 2(d)), and diabetes caused related cardiac damage, which was attenuated by rhGH treatment (Figure 2(e)).

### 3.3. rhGH Altered Cardiac Biomarker Activity and Prevented Cardiac Remodeling in db/db Mice

**Figure 3:**
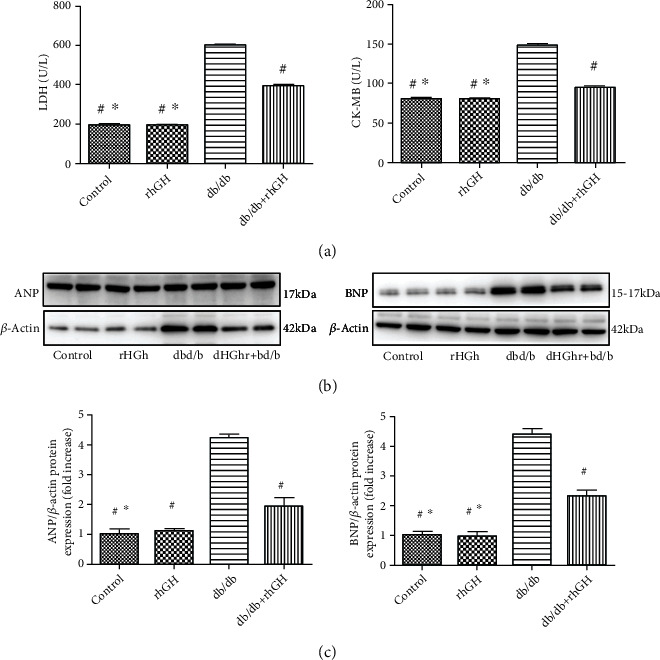
rhGH altered cardiac biomarker activity and prevented cardiac remodeling in db/db mice. (a) LDH and CK-MB levels were quantified using appropriate assay kits. *n* = 8 per group. ^∗^*P* < 0.05 vs. db/db+rhGH group and ^#^*P* < 0.01 vs. db/db group. (b) Expression of cardiac remodeling-associated biomarkers, including ANP and BNP. (c) Relative protein expression was quantified. *n* = 3 per group. ^∗^*P* < 0.05 vs. db/db+rhGH group and ^#^*P* < 0.01 vs. db/db group.

The activity of cardiac biomarkers, such as LDH and CK-MB, was significantly increased in db/db mice and decreased in db/db+rhGH mice (Figure 3(a)). The levels of cardiac remodeling-associated biomarkers, including ANP and BNP, were significantly increased in db/db mouse hearts and decreased by rhGH treatment (Figures 3(b) and 3(c)).

### 3.4. rhGH Improved Lipid Metabolism in db/db Mice

**Figure 4:**
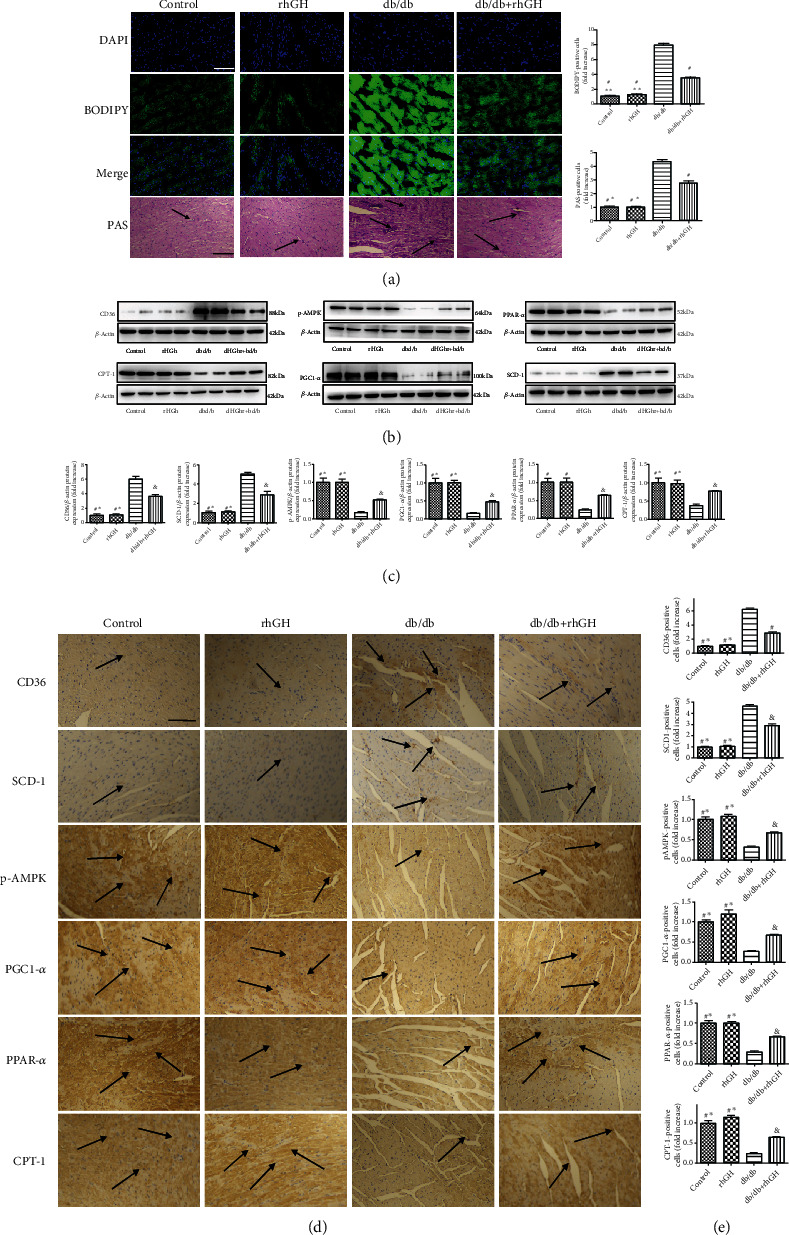
rhGH improved lipid metabolism in db/db mice. (a) BODIPY-stained (green fluorescence) and DAPI-stained (blue fluorescence) photomicrographs and PAS staining in the cardiac tissue. (b) Cardiac p-AMPK, CPT-1, PGC1-ɑ, PPAR-*α*, CD36, and SCD-1 levels were measured by western blotting. (c) Relative protein expression was quantified. (d) Representative immunohistochemistry for p-AMPK, CPT-1, PGC1-ɑ, PPAR-*α*, CD36, and SCD-1 in the cardiac tissues. (e) Positive expression was quantified. Scale bar: 100 *μ*m. The arrows indicate positively stained cells. *n* = 3 per group. ^∗^*P* < 0.05 vs. db/db+rhGH group, ^∗∗^*P* < 0.01 vs. db/db+rhGH group, ^&^*P* < 0.05 vs. db/db group, and ^#^*P* < 0.01 vs. db/db group.

To understand the mechanism underlying db/db-induced cardiac dysfunction and remodeling, we examined cardiac lipotoxicity. BODIPY and PAS staining showed lipid deposition of diabetic cardiomyopathy in db/db mouse heart, which was alleviated by rhGH treatment (Figure 4(a)). Moreover, we performed western blotting (Figures 4(b) and 4(c)) and immunohistochemistry (Figures 4(d) and 4(e)) to evaluate indicators related to lipid metabolism. We found that the phosphorylation of AMPK, CPT-1, PGC1-ɑ, and PPAR-*α* was significantly decreased in the db/db mouse heart tissue; rhGH restored p-AMPK, CPT-1, PGC1-ɑ, and PPAR-*α* expression. The expression of CD36 and SCD-1 significantly increased in the db/db group; however, their expression was downregulated by rhGH treatment. We obtained comparable results in the immunohistochemistry analysis.

### 3.5. rhGH Decreased Oxidative Stress in db/db Mice

**Figure 5:**
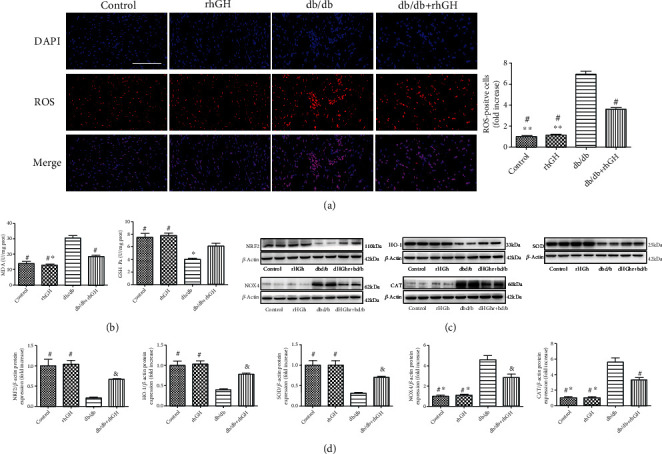
rhGH decreased oxidative stress in db/db mice. (a) ROS-stained (red fluorescence) and DAPI-stained (blue fluorescence) photomicrographs. Scale bar: 100 *μ*m, *n* = 3 per group. ^∗∗^*P* < 0.01 vs. db/db+rhGH group and ^#^*P* < 0.01 vs. db/db group. (b) Cardiac MDA and GSH activities were quantified using commercial assay kits. *n* = 8 per group. ^∗^*P* < 0.05 vs. db/db+rhGH group and ^#^*P* < 0.05 vs. db/db group. (c) Cardiac NRF2, HO-1, SOD, NOX4, and CAT levels were measured by western blotting. (d) Relative protein expression was quantified. *n* = 3 per group. ^∗^*P* < 0.05 vs. db/db+rhGH group, ^&^*P* < 0.05 vs. db/db group, and ^#^*P* < 0.01 vs. db/db group.

Oxidative stress plays a key role in the development of DCM. We used ROS staining to evaluate the expression of oxidative stress, and the number of ROS-positive areas was significantly increased in the heart of db/db mice; however, the increased were obviously reduced in db/db+rhGH mice (Figure 5(a)). The level of malondialdehyde (MDA), a key indicator of lipid peroxidation, was significantly higher in db/db mouse hearts, but the level of GSH (oxidative stress) was decreased. rhGH restored GSH level and decreased MDA level (Figure 5(b)). Moreover, we performed western blotting (Figures 5(c) and 5(d)) to evaluate the expression of indicators related to oxidative stress. The NRF2, HO-1, and SOD levels were significantly downregulated in the db/db mouse heart tissues, and rhGH restored these levels. Moreover, the expression of NOX4 and CAT significantly increased in the db/db group; however, their expression was downregulated by rhGH treatment.

### 3.6. rhGH Decreased Apoptosis in db/db Mice

**Figure 6:**
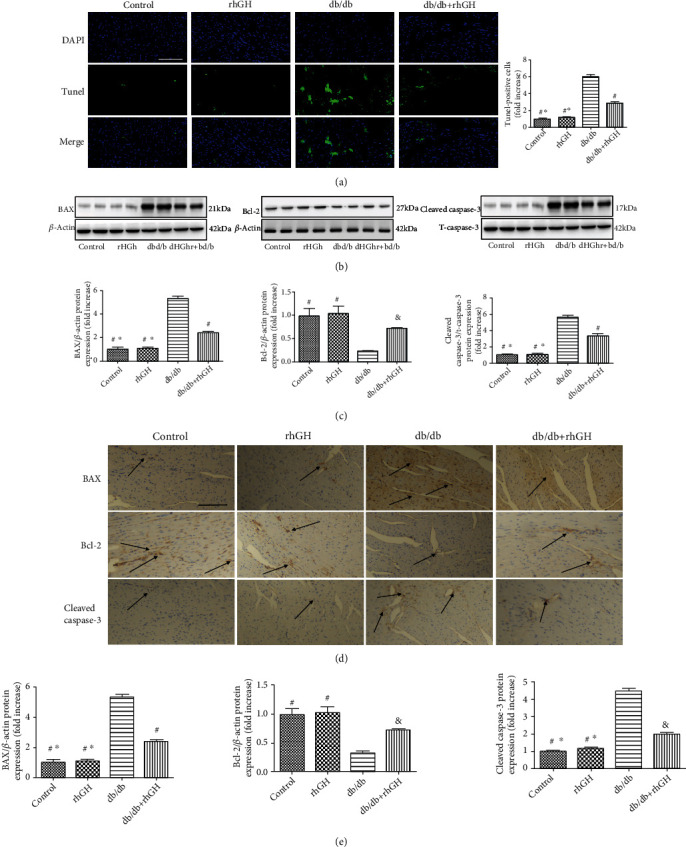
rhGH treatment decreased apoptosis in db/db mice. (a) TUNEL-stained (green fluorescence) and DAPI-stained (blue fluorescence) photomicrographs. (b) Cardiac BAX, Bcl-2, cleaved caspase-3, and t-caspase-3 levels were measured by western blotting. (c) Relative protein expression was quantified. (d) Representative immunohistochemistry for BAX, Bcl-2, and cleaved caspase-3 in the cardiac tissues. (e) Positive expression was quantified. Scale bar: 100 *μ*m. The arrows indicate positively stained cells. *n* = 3 per group. ^∗^*P* < 0.05 vs. db/db+rhGH group, ^&^*P* < 0.05 vs. db/db group, and ^#^*P* < 0.01 vs. db/db group.

Cardiomyocyte apoptosis is one of the main causes of structural defects in cardiac tissues in diabetes. The number of TUNEL-positive cells was significantly increased in the heart of db/db mice; however, the increased were obviously reduced in db/db+rhGH mice (Figure 6(a)). Immunoblotting (Figures 6(b) and 6(c)) and immunohistochemistry (Figures 6(d) and 6(e)) showed that in mice of the db/db group, the expression of the proapoptotic proteins BAX and cleaved caspase-3 was upregulated and that of Bcl-2 was downregulated, compared with those in mice of the control group. In addition, the rhGH treatment reduced the BAX and cleaved caspase-3 levels and upregulated Bcl-2 expression. These results indicate that rhGH has a beneficial effect on heart tissue in db/db mice.

## 4. Discussion

**Figure 7:**
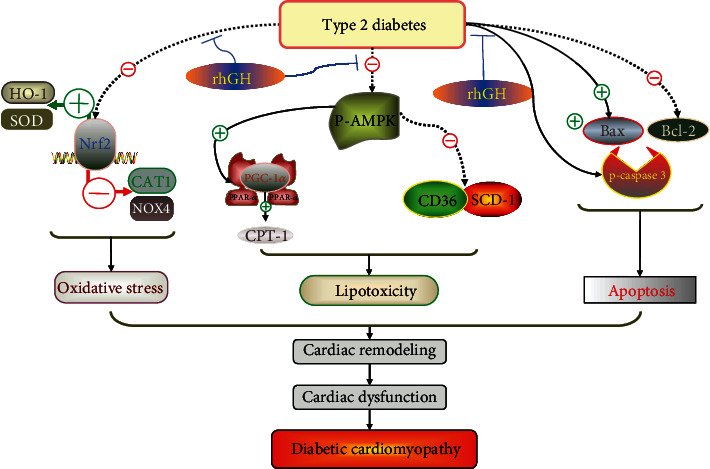
Schematic representation of how rhGH protects the heart against diabetic cardiomyopathy in db/db mice.

In this study, we observed decreased cardiac function and signs of myocardial injury in db/db mice. Typical pathological changes of DCM were also observed, including cardiac dysfunction, collagen deposition, lipid accumulation, oxidative damage, and apoptosis. Importantly, rhGH prevented cardiac dysfunction and reduced cardiac damage. These findings are summarized in Figure 7.

Our results show that administration of rhGH significantly improved DCM-induced systolic dysfunction and did not cause significant changes in blood glucose levels. Studies have shown that rhGH can improve the systolic function of the heart, while appropriate doses of rhGH have no effect on insulin resistance in rats [31, 32]. Consistently, data regarding the use of rhGH in adults and children for up to 10 years showed no adverse events related to diabetes [28]. It has been established that rhGH influences fat distribution; rhGH-replacement therapy administered to the hypopituitary adults decreases abdominal fat [33, 34]; rhGH also affects adipocyte metabolism by regulating fatty acid storage and release [35]. Our study showed that rhGH significantly affects the changes in lipid metabolism and reduces the body weight of db/db mice.

The accumulation of lipid droplets and lipid metabolites gradually deteriorates the structure and function of the myocardium [36] via lipotoxicity [37]. Lipotoxicity is more severe in T2DM than in T1DM and is a key feature in patients with T2DM [38]. Therefore, it is important to study whether rhGH can effectively reduce the lipid accumulation in the hearts of mice with T2DM. Zhou et al. showed that sodium-glucose cotransporter 2 alleviates diabetic heart injury by increasing AMPK phosphorylation [39]. CD36 is expressed on various cells as a scavenger receptor and has been shown to be a negative regulator of AMPK [36, 40]. We observed decreased AMPK activity in DCM throughout the study duration, while rhGH effectively restored AMPK phosphorylation (Figure 4(b)). The upregulated expression of activated AMPK, PPAR-*α*, PPAR-*γ* coactivator-1 alpha (PGC-1*α*), CPT-1, and downregulated activities of stearyl coenzyme A decarboxylase- (SCD-) 1 can improve dysregulated mitochondrial fatty acid (FA) metabolism and inhibit abnormal lipogenesis [41–44]. In this study, the expression of p-AMPK, CPT-1, PGC1-ɑ, and PPAR-*α* was significantly downregulated in the db/db mouse heart tissue, and rhGH treatment restored their expression. In contrast, the expression of CD36 and SCD-1 was significantly increased in the db/db group; however, it was downregulated following rhGH treatment.

Oxidative stress is intricately related to the development and progress of DCM [11]. Hyperglycemia can cause the overproduction of ROS, which subsequently, leads to excessive superoxide generation leading to cellular calcium overload. Collectively, increased oxidative stress and calcium overload promote high-glucose-induced cardiomyocyte death via apoptosis [45]. However, a few antioxidants are effective for DCM treatment. For instance, Nrf2 has been suggested as a potential target for various chronic diseases, including metabolic diseases, cardiovascular diseases, and neurodegenerative diseases [46]. Nrf2 induces the expression of a broad panel of antioxidant genes, such as those encoding glutathione-*S*-transferase, superoxide dismutase, and heme oxygenase-1 (HO-1), by binding to antioxidant response elements [46, 47]. Its expression is downregulated in the heart of animals and patients with diabetes. In experimental diabetes models, Nrf2 deficiency can increase oxidative and nitrosative stresses and cause early-stage cardiac damage and dysfunction [48]. Nrf2 deficiency in the diabetic model resulted in severe cardiac fibrosis and increased reactive oxygen species production. In the present study, we demonstrated that rhGH increased the expression of Nrf2, SOD, and HO-1 and decreased the expression of NOX4 and CAT. Taken together, our results indicate that rhGH can effectively induce Nrf2 expression to protect against DCM.

Apoptosis is also involved in the process of DCM [6]. The ratio of apoptotic cells was significantly reduced by rhGH treatment. The rhGH treatment decreased the expression of the proapoptotic proteins BAX and cleaved caspase-3 and increased the expression of the antiapoptotic protein Bcl-2 in mice with diabetes. Therefore, reducing apoptosis is one of the key mechanisms by which rhGH protects the heart against diabetic cardiomyopathy.

However, this study had a few limitations. First, we used only single dose for our experiments, and second, we did not estimate insulin levels, and this experiment needs to be confirmed in other animal models. Hence, our study outcomes need to be validated in future studies designed to overcome these limitations. Nevertheless, our study outcomes provide novel insights for therapeutic potential of rhGH.

## 5. Conclusions

In conclusion, our results suggest that rhGH treatment can protect against structural and functional changes in the cardiac tissue of db/db mice. In the future, rhGH may protect type 2 diabetes patients with cardiomyopathy by reducing lipotoxicity, oxidative stress, and apoptosis in clinical setting. Furthermore, the potential of GH in weight loss should be explored. Thus, rhGH is a promising agent for treating and preventing DCM.

## Data Availability

Data analyzed or generated during this study are included in this manuscript.
